# Towards Interpretable Seizure Detection: An Excitation/Inhibition Dynamic Polynomial Network Framework for Electroencephalography

**DOI:** 10.3390/s26113488

**Published:** 2026-06-01

**Authors:** Xihan Sun, Ying Yan, Na Liu, Shencun Fang, Jun Cai, Edmond Qi Wu, Aiguo Song, Junjie Xu

**Affiliations:** 1 Reading Academy, Nanjing University of Information Science and Technology, Nanjing 210044, China; mz804008@student.reading.ac.uk; 2 School of Automation, Jiangsu Engineering Research Center on Meteorological Energy Using and Control (C-MEIC), Jiangsu Collaborative Innovation Center of Atmospheric Environment and Equipment Technology (CICAEET), and State Key Laboratory of Environment Characteristics and Effects for Near-Space, Nanjing University of Information Science and Technology, Nanjing 210044, China; j.cai@nuist.edu.cn; 3 School of Mechanical and Electrical Engineering, Anhui Jianzhu University, Hefei 230009, China; 4 Department of Respiratory Medicine, The Affiliated Brain Hospital of Nanjing Medical University, Nanjing 210029, China; liuna860@njmu.edu.cn (N.L.); fang1984@aliyun.com (S.F.); 5 Department of Automation, Shanghai Jiao Tong University, Shanghai 200240, China; edmondqwu@sjtu.edu.cn; 6 School of Instrument Science and Engineering, Southeast University, Nanjing 210096, China; a.g.song@seu.edu.cn; 7 Waterford Institute, Nanjing University of Information Science and Technology, Nanjing 210044, China; 202383890027@nuist.edu.cn

**Keywords:** physics-informed neural networks, seizure detection, Wilson–Cowan dynamics, excitatory/inhibitory imbalance

## Abstract

Epilepsy is a prevalent neurological disorder characterized by recurrent seizures, and electroencephalogram (EEG) signals provide a direct measure of brain activity for detection. Although deep learning achieves high accuracy, it often lacks physiological interpretability. We propose the Excitation/Inhibition Dynamic Polynomial Network (E/I-DynPolyNet), a biologically grounded framework for interpretable seizure detection. Specifically, E/I-DynPolyNet introduces a dual excitatory/inhibitory (E/I) pathway with sign-constrained synaptic weights, encouraging the learned activations to reflect latent E/I representations. Furthermore, a differentiable Wilson-Cowan (WC) module is embedded to govern the temporal evolution of E/I interactions, ensuring consistency with neurophysiological principles. A physics-informed optimization strategy integrates supervised learning with dynamical residual constraints and E/I balance regularization, guiding the model to learn physiologically consistent representations. Experimental results on the CHB-MIT and Bonn datasets demonstrate competitive accuracies of 95.81% and 98.5%, respectively. Crucially, E/I-DynPolyNet enables quantitative estimation of E/I imbalance, revealing that E/I ratios increase from 1.01 in the pre-ictal phase to 1.38 during seizures—a finding consistent with clinical observations of ictogenesis. These results indicate that E/I-DynPolyNet not only improves detection performance but also provides a mechanistic description of seizure dynamics, bridging the gap between data-driven learning and neurophysiological interpretation.

## 1. Introduction

### 1.1. Background and Motivation

Epilepsy, a chronic neurological disorder affecting approximately 52 million people worldwide, ranks among the most prevalent non-communicable diseases in contemporary healthcare systems. It is characterized by recurrent and unpredictable seizures arising from excessive and hypersynchronous neuronal activity in the brain, imposing substantial medical, social, and economic burdens on patients and healthcare systems alike [1]. Therefore, accurate and reliable seizure detection is a fundamental task in clinical neurophysiology, directly affecting diagnosis, treatment planning, and long-term patient monitoring [2]. Electroencephalogram (EEG) signals, providing non-invasive measurements of brain electrical activity with high temporal resolution, remain the most widely used modality for capturing the dynamical signatures of epileptic events [3].

Deep learning has transformed EEG-based epilepsy detection by reducing the dependence on manual effort in feature engineering and improving discriminative performance. CNN-based models have been widely used to extract local spatial or spectral patterns from EEG segments [4,5]. Recurrent and hybrid CNN-RNN architectures further extend this capability by modeling temporal dependencies across consecutive signal windows [6,7]. Furthermore, recent advances in attention mechanisms and graph neural networks enable more sophisticated modeling of inter-channel dependencies and dynamic connectivity patterns [8,9]. These data-driven approaches consistently outperform traditional machine learning methods in terms of sensitivity, specificity, and computational efficiency [10]. Nevertheless, despite their impressive discriminative capabilities, most deep learning models remain black boxes, with limited transparency in their decisions and little insight into the neurophysiological mechanisms of seizure generation [11].

To address these limitations, recent studies have explored Physics-Informed Neural Networks (PINNs) and hybrid frameworks that incorporate domain knowledge from dynamical systems and computational neuroscience into deep learning models [12,13,14]. However, current PINN-based approaches in epilepsy detection remain fundamentally limited in three respects: First, many methods rely on simplified neural ordinary differential equation (ODE) models or generic constraints that do not adequately capture the complex nonlinear interactions underlying seizure generation. Second, neurophysiological models are often introduced as auxiliary regularizers or post hoc interpretability tools, rather than being structurally embedded into the learning architecture. Third, existing frameworks rarely establish a direct connection between learned representations and measurable neurophysiological quantities, thereby limiting their value in measurement-oriented analysis and clinical interpretation.

From a neurophysiological perspective, the excitation/inhibition (E/I) imbalance has long been recognized as a fundamental mechanism underlying epileptic seizures [15,16]. The pathological disruption of the balance between excitatory and inhibitory neuronal populations leads to abnormal network synchronization and seizure onset [17]. Among various modeling approaches, the Wilson–Cowan (WC) equations provide a well-established mathematical framework for describing the coupled dynamics of interacting excitatory and inhibitory populations, capturing key features such as nonlinear activation, saturation, and oscillatory behavior. Despite their strong biological interpretability and dynamical expressiveness, WC-based models have rarely been integrated into end-to-end learning frameworks for seizure detection in a principled and differentiable manner.

Existing attempts to incorporate neurophysiological knowledge into deep learning models suffer from several critical limitations. On the one hand, simplified neural mass models or linear approximations fail to capture the asymmetric, nonlinear, and time-dependent interactions between excitatory and inhibitory populations observed in real cortical systems [18,19]. On the other hand, approaches that treat E/I dynamics as post hoc analysis tools do not allow the model to leverage physics-based constraints during representation learning, resulting in suboptimal generalization and limited physiological consistency [20]. Furthermore, the temporal evolution of E/I imbalance exhibits characteristic dynamical patterns with distinct time constants, which require explicit modeling within the computational graph rather than indirect inference. Although scalp EEG reflects mixed activity from heterogeneous neural populations, seizure-related dynamics can still be described from a neural population perspective. In this view, the WC model provides a mesoscopic formulation for describing interactions between excitatory and inhibitory populations rather than individual neurons. Therefore, for scalp EEG-based seizure recognition, we model large-scale neural activity using population-level excitatory and inhibitory population variables.

### 1.2. Novelty Relative to Existing Physics-Informed EEG Frameworks

Although neurophysiological and dynamical priors have been explored in EEG learning, their roles in existing frameworks differ from that in E/I-DynPolyNet. Neural mass model-based EEG learning systems usually describe population-level brain activity through mechanistic dynamical equations. However, they are more commonly used for simulation, parameter fitting, or mechanistic interpretation, rather than serving as trainable internal modules in an end-to-end EEG classifier. Existing WC-inspired neural architectures can model interactions between excitatory and inhibitory populations with strong physiological interpretability, but they often focus on standalone dynamical modeling and do not explicitly couple WC-governed E/I latent states with deep temporal feature learning for seizure state discrimination. Physics-informed EEG classifiers typically introduce physiological or differential equation constraints as auxiliary residual losses, while the learned latent representation itself remains largely abstract. Biologically constrained RNNs improve temporal modeling through recurrent, activation, or connectivity constraints, but they usually do not provide an explicit E/I pathway decomposition governed by WC population dynamics.

E/I-DynPolyNet differs from these approaches by embedding the E/I prior into the internal architecture of the classifier. The temporal EEG representation is first projected into excitatory and inhibitory pathways, and these latent population-like states are then coupled through a differentiable WC dynamic module. In this way, the model constrains representation learning through explicit E/I population interactions rather than relying only on abstract latent features or external physics-informed penalties.

The polynomial expansion and WC dynamics are designed to fulfill complementary functional roles. Specifically, the lightweight quadratic expansion enriches nonlinear feature representation in a computationally efficient manner, while the WC module enforces physiologically grounded E/I interaction constraints on the latent neural dynamics. Consequently, the proposed model unifies nonlinear feature augmentation, temporal encoding, E/I decomposition, and neurodynamic regularization within a single, end-to-end trainable framework for seizure classification.

The main contributions of this work can be characterized as follows:

- We introduce a physics-informed polynomial layer that embeds interpretable dynamical properties, drawing on the fact that physically meaningful differential equations become polynomial forms after Laplace transformation.
- We design a structural-level physics-informed architecture that realizes explicit excitation/inhibition decoupling. By hard-coding the E/I interaction mechanism into the network topology, we simulate the segregated processing of cortical populations with guaranteed mechanistic consistency.
- We achieve strong seizure detection accuracy on the CHB-MIT and Bonn EEG datasets, and we quantitatively reveal seizure-associated E/I imbalance in agreement with clinical observations.
- We verify through ablation studies that both E/I separation and temporal encoding are essential components, showing clear advantages over conventional, purely data-driven baselines.

The remainder of this paper is organized as follows: Section 2 describes the proposed E/I-DynPolyNet, including the polynomial expansion layer, causal temporal encoding, dual E/I pathways, WC dynamics module, and composite optimization objective; Section 3 presents the EEG-based epilepsy diagnosis pipeline, with datasets (CHB-MIT and Bonn), preprocessing, training protocol, and evaluation metrics; Section 4 reports the experimental results, including classification performance, E/I imbalance visualization, and ablation analyses; Section 5 provides a comparative evaluation against representative baseline models; and Section 6 concludes the paper, with limitations and future research directions.

## 2. E/I-DynPolyNet Model

### 2.1. The Structure of the E/I-DynPolyNet

**Figure 1:**
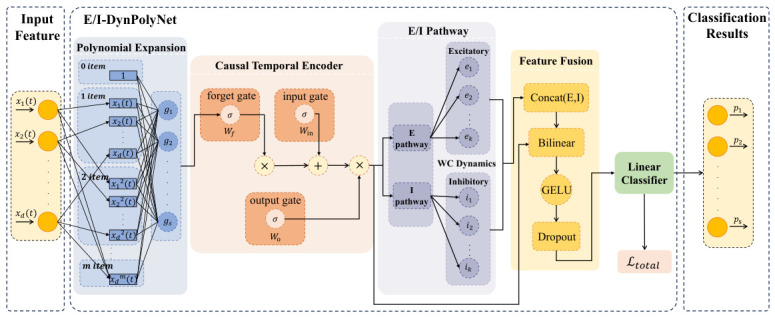
Structural diagram of the E/I-DynPolyNet classification model.

The proposed E/I-DynPolyNet classification model architecture primarily comprises a polynomial expansion layer, causal temporal encoding, an E/I dual-path mechanism, and feature fusion, as illustrated in the structural diagram shown in Figure 1.

In this model, $x_{i}\left(t\right)$ denotes the raw input feature of channel *i* at time step *t*, the vector $x\left(t\right)={\left[x_{1}\left(t\right),x_{2}\left(t\right),\ldots ,x_{d}\left(t\right)\right]}^{T}$ forms the input at time *t*, and $g_{s}$ signifies the *s*-th nonlinear feature dimension output from the polynomial expansion layer. $W_{f},W_{in}$, and $W_{o}$ denote weight matrices for the forget gate, input gate, and output gate within the temporal encoding layer, respectively; σ denotes the sigmoid activation function utilized for gate control; $e_{k}$ and $i_{k}$ denote the latent activation states of the *k*-th unit within the E pathway and I pathway, respectively; WC Dynamics denotes the WC neural dynamics module; $p_{n}$ denotes the final classification result; and $L_{\mathrm{total}}$ measures the discrepancy between the model output and the ground-truth label. A cosine annealing learning-rate schedule with warmup is employed to facilitate stable convergence during end-to-end optimization.

### 2.2. Polynomial Expansion Layer

The polynomial expansion layer approximates nonlinear mapping relationships of input features by using power series expansion, thus improving the representation of local features to help provide richer feature representations for the temporal modeling.

For the EEG signal input, $x_{i}\left(t\right)$ denotes the observation of the *i*-th feature channel at time *t*. The original input feature vector is defined as

(1) $$x\left(t\right)={\left[x_{1}\left(t\right),x_{2}\left(t\right),\ldots ,x_{d}\left(t\right)\right]}^{T},$$

where *t* denotes the time index and *d* represents the input feature dimension.

A full second-order expansion includes a constant term, all linear terms, and all quadratic cross-terms. The corresponding feature dimension becomes $n_{\mathrm{poly}}=1+d+\frac{d(d+1)}{2}$, which grows combinatorially with *d*. This high-dimensional representation creates a lot of computational overhead and, thus, is not implemented in this work.

To maintain computational efficiency while preserving nonlinear expressiveness, we employ a lightweight degree-2 polynomial mapping that retains only the diagonal quadratic terms. The expanded feature vector is defined as

(2) $$x^{\mathrm{poly}}\left(t\right)={\left[x_{1}\left(t\right),x_{2}\left(t\right),\ldots ,x_{d}\left(t\right),x_{1}^{2}\left(t\right),\ldots ,x_{d}^{2}\left(t\right)\right]}^{T}.$$

This compact representation can be equivalently written as

(3) $$x^{\mathrm{poly}}\left(t\right)=\left[x\left(t\right);x^{⊙2}\left(t\right)\right].$$

Here, $x^{⊙2}\left(t\right)$ denotes an element-wise square sequence, where the superscript ⊙2 denotes element-wise squaring and $\left[\;;\;\right]$ denotes vector concatenation.

After applying the lightweight degree-2 mapping, the resulting polynomial matrix $X^{\mathrm{poly}}$ is

(4) $$X^{\mathrm{poly}}\left(t\right)=\left[\begin{array}{cc} x_{1}\left(t\right), & x_{1}^{2}\left(t\right)\\ x_{2}\left(t\right), & x_{2}^{2}\left(t\right)\\ \vdots & \vdots \\ x_{d}\left(t\right), & x_{d}^{2}\left(t\right) \end{array}\right].$$

Here, $X^{\mathrm{poly}}$ denotes the polynomial matrix, where each node in the polynomial expansion layer is annotated as ${x_{i}}^{m}$. Accordingly, the dimensionality of the polynomial matrix $X^{\mathrm{poly}}$ is d×2, which is significantly smaller than the full second-order expansion.

It should be noted that the retained quadratic terms are diagonal, channel-wise terms rather than explicit pairwise cross-channel products. Therefore, they are not intended to encode all pairwise interactions. Instead, the diagonal quadratic expansion provides a compact nonlinear input basis that captures amplitude-dependent channel-wise effects while avoiding the rapid dimensional growth of a full second-order expansion. The cross-channel nonlinearities that are important for seizure propagation are therefore not modeled solely by the polynomial expansion layer but are further captured by the subsequent modules.

Although the squaring operation may amplify high-amplitude artifacts and potentially degrade the signal-to-noise ratio (SNR), this risk is mitigated by the preprocessing bandpass filtering and by the subsequent gating mechanisms, which help suppress non-physiological noise components during feature fusion.

### 2.3. Causal Temporal Encoder

An epileptic seizure is a complex neurodynamic process with distinct temporal and spatial features. Accurate seizure detection requires capturing the intrinsic temporal dependencies in the EEG signals, which show gradual evolution and history-dependent dynamics of seizures. By capturing such temporal information, the causal temporal encoder can build a stable gating and recurrent structure to learn long- and short-term temporal patterns followed, and to extract features that are smooth, temporally coherent, and aligned with the physics-informed E/I modeling requirements. In addition to the attention-based encoder, LSTM can lead to more stable training under physical regularization. Given the polynomial-expanded feature sequence $x^{\mathrm{poly}}\left(t\right),\;\;t=1,2,\ldots T$, the hidden state h(0) and memory cell C(0) are initialized as zero vectors. The module is composed of six submodules, defined as follows:

#### 2.3.1. State Layer Mapping

The polynomial-expanded input is first projected into the hidden space through

(5) $$s\left(t\right)=\tanh\left(W_{c}\cdot x^{\mathrm{poly}}\left(t\right)+U_{c}\cdot h_{t-1}+b_{c}\right),$$

where $W_{c}$ and $U_{c}$ are the input and recurrent weight matrices, respectively. The tanh(·) activation bounds the output in [−1, 1], stabilizing gradient propagation, and effectively mitigating gradient vanishing and exploding issues while accelerating convergence.

#### 2.3.2. Input Gate

The input gate governs the amount of new candidate information written to a cell state, consequently regulating the state update and filtering out irrelevant or noise inputs. It is defined according to

(6) $$i\left(t\right)=\sigma \left(W_{\mathrm{in}}\cdot x^{\mathrm{poly}}\left(t\right)+U_{\mathrm{in}}\cdot h_{t-1}+b_{\mathrm{in}}\right),$$

where i(t) denotes input gate activation, $W_{\mathrm{in}}$ represents the input gate parameter matrix, $U_{\mathrm{in}}$ is the recurrent weight matrix specifically for the input gate, and σ(·) is the sigmoid activation function $\sigma \left(x\right)=\frac{1}{1+e^{-x}}$, which maps the input to the interval [0, 1] that produces gating coefficients.

#### 2.3.3. Forget Gate

The forget gate determines which information from the previous cell state should be retained or discarded, increasing the adaptability of the model by changing the level of information preservation capability with a dynamic approach. The sigmoid function also has a similar approach, which outputs scores between 0 and 1 to describe the degree of information retention:

(7) $$f\left(t\right)=\sigma \left(W_{f}\cdot x^{\mathrm{poly}}\left(t\right)+U_{f}\cdot h_{t-1}+b_{f}\right),$$

where f(t) denotes the activation value of the forget gate, while $W_{f}$ and $U_{f}$ are the weight matrices controlling the level of information preservation.

#### 2.3.4. Output Gate

The output gate controls the extent to which information from memory cells is transmitted, effectively preventing feature redundancy and enhancing robustness:

(8) $$o\left(t\right)=\sigma \left(W_{o}\cdot x^{\mathrm{poly}}\left(t\right)+U_{o}\cdot h_{t-1}+b_{o}\right),$$

where o(t) represents the activation value of the output gate, while $W_{o}$ and $U_{o}$ denote the weight matrices of the output gate.

#### 2.3.5. Memory Cell Update

This module results from retaining part of the previous state through the forget gate and adding new candidate content through the input gate. This fusion of historical context and incoming information improves the model’s effectiveness in handling sequential data. The corresponding expression is

(9) C(t)=i(t)⊙s(t)+f(t)⊙C(t−1).

Here, ⊙ represents element-wise multiplication, C(t) is the memory state at time step *t*, and C(t−1) denotes the previous memory state.

#### 2.3.6. Hidden State Output

Finally, the hidden state is computed as follows:

(10) $$h\left(t\right)=o\left(t\right)⊙\tanh\left(C(t)\right),\;t=1,2,\ldots ,T.$$

This hidden state provides the temporally encoded representation used by the subsequent E/I pathway dynamics. The resulting hidden sequence h(t) is then used as the temporal representation for the subsequent E/I pathways. In addition, the encoded temporal context is used for conditional WC parameter generation and hybrid temporal pooling in the later feature fusion stage.

### 2.4. E/I Pathway

**Figure 2:**
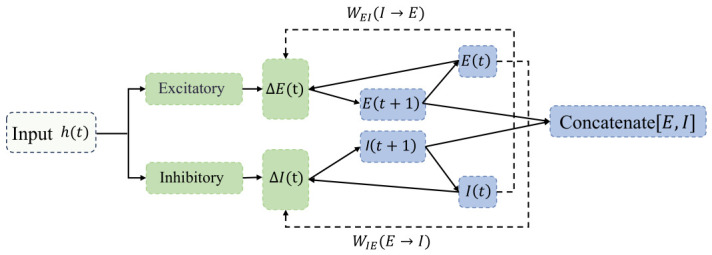
E/I pathway schematic diagram.

To address the limitations of black-box EEG classifiers, we propose a physics-informed dual-path architecture grounded in the WC neuronal population model. The network explicitly separates excitatory and inhibitory processing, embedding neurophysiological priors into the latent representation. By applying WC-based regularization to E/I states extracted from multi-channel EEG representations, the model constrains the learned latent dynamics with neurophysiological priors, while preserving the discriminative information encoded by the temporal feature extractor. The resulting E/I ratio also serves as a model-derived interpretable indicator associated with epileptic seizures. The general information flow and functional roles of the proposed module are illustrated in Figure 2.

#### 2.4.1. E-Pathway

The E-Pathway is modeled by neurons that increase the postsynaptic potential through positive activation. To ensure non-negative outputs that are consistent with the excitatory nature of pyramidal neurons, the study employs ReLU activation and defines as follows:

(11) $$E\left(t\right)=\mathrm{ReLU}\left(\mathrm{LN}\left(W_{E}\left[h\left(t\right),h{\left(t\right)}^{2}\right]+b_{E}\right)+R_{E}\left(h(t)\right)\right),$$

where h(t) denotes the LSTM output at time *t*, $W_{E}$ is the positive weight matrix, $b_{E}$ represents the bias term, LN(·) denotes layer normalization, and $R_{E}\left(\cdot \right)$ is a residual adapter ensuring dimensional compatibility.

#### 2.4.2. I-Pathway

The I-Pathway captures interneuron suppression through a signed transformation. Using the same augmented input $\left[h\left(t\right),{h(t)}^{2}\right]$, we define

(12) $$I\left(t\right)=\tanh\left(W_{I2}\tanh\left(\mathrm{LN}\left(W_{I1}\left[h\left(t\right),h{\left(t\right)}^{2}\right]+b_{I1}\right)+b_{I2}\right)\right)+R_{I}\left(h(t)\right),$$

where $W_{I1}$ and $W_{I2}$ are the weight matrices for the two-layer inhibitory network, with $b_{I1}$ and $b_{I2}$ as bias terms; tanh(·) activations enable bidirectional signal output characteristic of inhibitory neurons; and $R_{I}\left(h(t)\right)$ is a residual adapter ensuring dimensional compatibility.

#### 2.4.3. E/I Dynamic Interaction

The outputs from both pathways are coupled through a WC dynamic module:

(13) $$\left\{\begin{array}{cc} \Delta E(t) & =\frac{-E\left(t\right)+S\left(W_{EE}E\left(t\right)-W_{EI}I\left(t\right)\right)}{\tau_{E}}\cdot \Delta t,\\ \Delta I(t) & =\frac{-I\left(t\right)+S\left(W_{IE}E\left(t\right)-W_{II}I\left(t\right)\right)}{\tau_{I}}\cdot \Delta t, \end{array}\right.$$

where S(·) is a sigmoid activation function; $W_{EE},W_{EI},W_{IE}$, and $W_{II}$ denote synaptic coupling weights for self-excitation, cross-inhibition, excitatory-to-inhibitory, and self-inhibition connections, respectively; and $\tau_{E}$ and $\tau_{I}$ represent the time constants for each population.

Consistent with the population-level formulation of the WC model, the dynamic module operates on latent E/I states derived from multi-channel temporal features, so the learned coefficients describe effective excitatory/inhibitory interactions at the representation level, rather than spatial links among individual electrodes.

For implementation, the continuous-time WC dynamics are discretized using the explicit Euler method. For a general ODE dX(t)/dt=f(X(t)), the Euler approximation is given by $X^{k+1}=X^{k}+\Delta tf\left(X^{k}\right)$, where *k* denotes the internal evolution step. In experiments, the internal step size was set to Δt=0.1, and the WC module was iterated for K=8 evolution steps, corresponding to a fixed normalized integration horizon of KΔt=0.8.

The discrete evolution is conducted over a fixed number of WC steps, which limits long-horizon numerical accumulation and supports controlled latent-state propagation. Stability is further promoted by the bounded sigmoid response, positive time constants, and physics-informed residual regularization. The behavior of the discretized WC module is empirically monitored by tracking the physical residual during training.

After WC dynamic evolution, the excitatory and inhibitory latent states are temporally aggregated to compute a segment-level E/I ratio. This ratio is defined on the aggregated latent representation of an EEG segment, rather than on individual EEG channels. Therefore, the global E/I target does not imply identical E/I values across channels or local neural sources. Instead, it serves as a regularizer that encourages non-ictal samples to remain close to E/I balance while promoting excitation-dominant dynamics in ictal samples.

### 2.5. Feature Fusion

The E/I-DynPolyNet model integrates a gated fusion mechanism rather than simple concatenation to facilitate deep nonlinear interactions between the physical features derived from the E/I pathways and the temporal context captured by the causal temporal encoder [21]. Specifically, for each pathway, performing multidimensional feature extraction and fusion on the time series E(t) and I(t) yields

(14) $$\left\{\begin{array}{c} z_{E}\left(t\right)=\mathrm{MLP}\left(\mathrm{attn}\left(E_{T}\left(t\right)\right);\mathrm{mean}\left(E_{T}\left(t\right)\right);\max\left(E_{T}\left(t\right)\right);\mathrm{last}\left(E_{T}\left(t\right)\right)\right),\\ z_{I}\left(t\right)=\mathrm{MLP}\left(\mathrm{attn}\left(I_{T}\left(t\right)\right);\mathrm{mean}\left(I_{T}\left(t\right)\right);\max\left(I_{T}\left(t\right)\right);\mathrm{last}\left(I_{T}\left(t\right)\right)\right), \end{array}\right.$$

where $z_{E}\left(t\right)$ and $z_{I}\left(t\right)$ represent the aggregated excitatory and inhibitory features. For each pathway k∈{E,I}, $k_{T}\left(t\right)$ denotes the local observation sequence ending at physical time *t* with a time span of *T*, where $\mathrm{attn}\left(k_{T}\left(t\right)\right)$ is the weighted sum derived from a multi-head attention mechanism, while $\mathrm{mean}\left(k_{T}\left(t\right)\right)$, $\max\left(k_{T}\left(t\right)\right)$, and $\max\left(k_{T}\left(t\right)\right)$ capture the average activation, peak discharge, and steady characteristics of the neural population, respectively. Then, they are concatenated to form the integrated fusion vector:

(15) $$z_{EI}\left(t\right)=\left[z_{E}\left(t\right);z_{I}\left(t\right)\right].$$

$z_{EI}\left(t\right)$ is then passed through a nonlinear interaction gate, enabling the selection of physical states that are capable of modulating timing characteristics; it is computed as follows:

(16) $$g_{\mathrm{phys}}\left(t\right)=\sigma \left(\mathrm{LN}\left(W_{\mathrm{gate}}\cdot z_{EI}\left(t\right)+b_{\mathrm{gate}}\right)\right),$$

(17) $$h_{\mathrm{lstm}}\left(t\right)=\mathrm{HybridPool}\left(h(t)\right)⊙g_{\mathrm{phys}}\left(t\right),$$

where $h_{\mathrm{lstm}}\left(t\right)$ denotes the aggregated temporal features from the causal temporal encoder module, and LN(·) represents layer normalization.

Ultimately, a bilinear interaction layer is introduced to capture high-order correlations between the gated physical and temporal vectors:

(18) $$F_{\mathrm{bi}}\left(t\right)=h_{\mathrm{lstm}}\left(t\right)\cdot W_{\mathrm{bi}}\cdot z_{EI}^{⊺}\left(t\right),$$

(19) $$F_{\mathrm{res}}\left(t\right)=W_{\mathrm{res}}\left[h_{\mathrm{lstm}}\left(t\right);z_{EI}\left(t\right)\right]+b_{\mathrm{res}},$$

(20) $$F^{\prime }\left(t\right)=\mathrm{GELU}\left(W_{1}\left[F_{\mathrm{bi}}\left(t\right);F_{\mathrm{res}}\left(t\right)\right]+b_{1}\right),$$

where $F_{\mathrm{bi}}\left(t\right)$ represents the features resulting from the coupling of physical dynamics with temporal context. $W_{\mathrm{res}}$ denotes the residual projection matrix, which is responsible for linearly mapping the original features after stitching into the fusion space, while b_res_ denotes the residual term bias. GELU(·) denotes the Gaussian Error Linear Unit, a smooth nonlinear activation function. Finally, through layer normalization and dropout processing, the final features $F^{\prime \prime }\left(t\right)$ are generated:

(21) $$F^{\prime \prime }\left(t\right)=\mathrm{LN}\left(F^{\prime }\left(t\right)\right)+\mathrm{Dropout}\left(F^{\prime }\left(t\right)\right),$$

where $F^{\prime \prime }\left(t\right)$ denotes the final unified classification characterization.

### 2.6. Classification Layer

Subsequent to feature fusion, the ultimate representation, designated $F^{\prime \prime }\left(t\right)$, is mapped to the decision space via a fully connected classification layer. The resulting logits are then fed into a softmax function to yield the predicted class-probability vector.

The classification process first undergoes a hidden-layer transformation:

(22) $$F_{\mathrm{cls}}\left(t\right)=\mathrm{Dropout}\left(\mathrm{GELU}\left(W_{C1}F^{\prime \prime }\left(t\right)+b_{C1}\right)\right).$$

Here, $W_{C1}$ and $b_{C1}$ represent the weight matrix and bias vector of the first fully connected layer, respectively. Then, $F_{\mathrm{cls}}\left(t\right)$ is projected onto the output logits z(t) for the target class:

(23) $$z\left(t\right)=W_{C2}\cdot F_{\mathrm{cls}}\left(t\right)+b_{C2},$$

where $W_{C2}$ maps the features to the corresponding output class. The softmax function transforms the logits into a normalized probability distribution, where the i-th element ${\hat{y}}_{i}\left(t\right)$ is computed as

(24) $${\hat{y}}_{i}\left(t\right)=\frac{e^{z_{i}\left(t\right)}}{\sum_{j=1}^{C}e^{z_{j}\left(t\right)}}.$$

Here, $z_{i}\left(t\right)$ denotes the i-th logit produced by the classifier at time *t*, while *j* indexes all classes in the normalization term.

### 2.7. Parameter Learning of the E/I-DynPolyNet Model

The parameter learning process of the proposed E/I-DynPolyNet integrates data-driven supervision and physics-guided regularization within a unified optimization framework. The model parameters are defined as follows:

(25) $$W=\left\{W_{E},W_{I},W_{WC},W_{\mathrm{fuse}},W_{\mathrm{cls}}\right\},$$

where $W_{E}$ and $W_{I}$ represent the dual pathways’ weights and biases $\left\{W_{E},b_{E}\right\},\left\{W_{I1},W_{I2},b_{I1},b_{I2}\right\}$. $W_{WC}$ represents the WC coupling coefficients $\left\{W_{EE},W_{EI},W_{IE},W_{II}\right\}$. $W_{\mathrm{fuse}}$ and $W_{\mathrm{cls}}$ represent the weights of the fusion layer $\left\{W_{\mathrm{gate}},W_{\mathrm{bi}},W_{\mathrm{res}},W_{1}\right\}$ and classification layer $\left\{W_{C1},W_{C2}\right\}$, respectively.

During training, E/I-DynPolyNet minimizes a composite objective function combining statistical and biophysical components

(26) $$L_{\mathrm{total}}=L_{\mathrm{CE}}+\lambda_{\mathrm{phy}}L_{\mathrm{phy}}+\lambda_{\mathrm{bal}}L_{\mathrm{bal}}+\lambda_{\mathrm{reg}}L_{\mathrm{reg}}$$

where $\lambda_{\mathrm{phy}},\lambda_{\mathrm{bal}},$ and $\lambda_{\mathrm{reg}}$ are non-negative scalars that balance the contributions of physics residual, E/I equilibrium, and weight regularization, respectively. To prevent the physics constraints from being dominated by other terms in the total loss, $\lambda_{\mathrm{phy}}$ is a dynamic weight following a warmup schedule, which ensures that the physics residual term contributes significantly to the total loss without compromising classification performance [22]. Neurophysiological research shows that healthy neural circuitry operates at the current ratio of cortical excitatory and inhibitory functions, and that an imbalance will lead to abnormal neurodynamic or functional impairment [23]. Therefore, setting $\lambda_{\mathrm{bal}}=0.005$ enables the model to maintain biological consistency while avoiding significant interference with classification learning gradients [24]. $\lambda_{\mathrm{reg}}=0.0001$ employs the empirical value employed in deep learning. This value attains the proper trade-off between avoiding overfitting and maintaining model expressiveness, and it matches the normal practice of L2 regularization in deep learning [25]. With this multi-level hyperparameter design, the E/I-DynPolyNet model achieves good and stable performance for the classification of EEGs while meeting the constraints of neuroscientific physics.

In this framework, the physics-guided terms are designed to provide auxiliary constraints on E/I dynamics, while the supervised classification objective remains the main driving force of parameter learning. The WC-based residual therefore acts as a physiological regularizer that encourages dynamically consistent E/I evolution, rather than as an independent simulator that determines the final prediction alone. Accordingly, the contribution of the physical constraint needs to be balanced with the discriminative objective. An excessively large $\lambda_{\mathrm{phy}}$ or an overly long WC evolution horizon may force the latent states to conform too strongly to idealized neural population dynamics, reducing the representational flexibility required for heterogeneous EEG patterns. From this perspective, the transition from a physics-informed classifier to a simulator-like model depends on whether the dominant objective is discriminative accuracy or trajectory-level dynamical fidelity. In the present model, the former remains the primary objective, and the WC equations serve to regularize and interpret the learned E/I representation.

#### 2.7.1. Joint Gradient Computation

For each parameter subset $W_{k}\in W$, the total gradient is expressed as follows:

(27) $$\nabla_{W_{k}}L_{\mathrm{total}}=\nabla_{W_{k}}L_{\mathrm{CE}}+\lambda_{\mathrm{phy}}\nabla_{W_{k}}L_{\mathrm{phy}}+\lambda_{\mathrm{bal}}\nabla_{W_{k}}L_{\mathrm{bal}}+\lambda_{\mathrm{reg}}\nabla_{W_{k}}L_{\mathrm{reg}}.$$

All parameters share the same gradient framework, differing only in the extent to which different modules are affected by different loss terms. The physical residual term introduces additional gradients with respect to the WC coupling parameters. The theoretical finite-step increment derived from the WC equation is as follows:

(28) $$\left\{\begin{array}{c} \Delta E{\left(t\right)}_{theory}=\frac{-E\left(t\right)+\sigma \left(W_{EE}E\left(t\right)-W_{EI}I\left(t\right)\right)}{\tau_{E}}\cdot \Delta t,\\ \Delta I{\left(t\right)}_{theory}=\frac{-I\left(t\right)+\sigma \left(W_{IE}E\left(t\right)-W_{II}I\left(t\right)\right)}{\tau_{I}}\cdot \Delta t, \end{array}\right.$$

where σ(·) denotes the sigmoid function.

The predicted finite-step increments provided by the state transition network are denoted as $\Delta E_{\mathrm{net}}\left(t\right)$ and $\Delta I_{\mathrm{net}}\left(t\right)$, and its physical residual is defined as follows:

(29) $$L_{\mathrm{phy}}=\frac{1}{T}\sum_{t=1}^{T}\left({∥\Delta E_{\mathrm{net}}\left(t\right)-\Delta E{\left(t\right)}_{\mathrm{theory}}∥}^{2}+{∥\Delta I_{\mathrm{net}}\left(t\right)-\Delta I{\left(t\right)}_{\mathrm{theory}}∥}^{2}\right).$$

Then, successively taking the derivative with respect to each element-wise parameter $W_{pq,d}$(p,q∈{E,I}) of $W_{\mathrm{WC}}$ yields

(30) $$\frac{\partial L_{\mathrm{phy}}}{\partial W_{pq,d}}=\frac{2}{T}\sum_{t=1}^{T}\left(\Delta X_{p,d}{\left(t\right)}_{\mathrm{theory}}-\Delta X_{p,d}{\left(t\right)}_{\mathrm{net}}\right)\frac{\partial \Delta X_{p,d}{\left(t\right)}_{\mathrm{theory}}}{\partial W_{pq,d}},$$

The derivative term is given by

(31) $$\frac{\partial \Delta X_{p,d}{\left(t\right)}_{\mathrm{theory}}}{\partial W_{pq,d}}=\frac{\Delta t}{\tau_{p}}\sigma^{\prime }\left(W_{pE,d}E_{d}\left(t\right)-W_{pI,d}I_{d}\left(t\right)\right)s_{q}X_{q,d}\left(t\right),\;p,q\in \left\{E,I\right\},$$

where $X_{E}\left(t\right)=E\left(t\right)$, $X_{I}\left(t\right)=I\left(t\right)$, *d* denotes the latent E/I dimension, and $s_{E}=+1$, $s_{I}=-1$.

#### 2.7.2. Adaptive Moment Estimation for Multi-Scale Gradients

Considering the different gradient magnitudes introduced by different activation functions, recurrent temporal encoding, E/I pathway decomposition, and WC-based physical residuals, E/I-DynPolyNet employs the AdamW optimizer to stabilize multi-module optimization.

At iteration *i*, the corresponding first and second moment statistics of $g_{i}=\nabla_{\Theta_{i}}L_{\mathrm{total}}$ can be computed as follows:

(32) $$m_{i}=\beta_{1}m_{i-1}+\left(1-\beta_{1}\right)g_{i},\;v_{i}=\beta_{2}v_{i-1}+\left(1-\beta_{2}\right)g_{i}^{2}$$

Bias-corrected estimates are obtained as follows:

(33) $${\hat{m}}_{i}=\frac{m_{i}}{1-\beta_{1}^{i}},\;{\hat{v}}_{i}=\frac{v_{i}}{1-\beta_{2}^{i}}.$$

The parameter update rule becomes

(34) $$\Theta_{i}=\Theta_{i-1}-\eta_{i}\left(\frac{{\hat{m}}_{i}}{\sqrt{{\hat{v}}_{i}}+\epsilon }+\lambda \cdot \Theta_{i-1}\right),$$

where $\eta_{i}$ is the adaptive learning rate, λ is the weight decay coefficient, and ϵ is a small stabilizing constant.

Adam’s moment estimation effectively balances the gradient magnitudes of the E pathway and I pathway, preventing excitatory dominance or inhibitory collapse during training.

#### 2.7.3. Learning-Rate Scheduling and Stability Control

A strategy of cosine annealing with warmup is incorporated for dynamic learning-rate scheduling:

(35) $$\eta \left(i\right)=\eta_{\min}+\frac{1}{2}\left(\eta_{\max}-\eta_{\min}\right)\left(1+\cos\left(\frac{i-T_{\mathrm{warm}}}{T_{\mathrm{total}}-T_{\mathrm{warm}}}\pi \right)\right),$$

where η(i) is the effective learning rate at the current epoch *i*, while $\eta_{\max}$ and $\eta_{\min}$ denote the maximum and minimum learning rate, respectively. $T_{\mathrm{warm}}$ is set to 6, representing the number of warmup epochs, and $T_{\mathrm{total}}$ is the total number of training epochs.

Furthermore, gradient clipping is enforced to mitigate instability from large physical residuals:

(36) $$∥\nabla_{\Theta_{i}}{∥}_{2}\leq 1.0.$$

#### 2.7.4. Biophysical Parameter Adaptation

The $W_{WC}$ is iteratively updated as follows:

(37) $$w_{pq}^{i+1}=w_{pq}^{i}-\eta \left(i\right)\frac{\partial L_{\mathrm{total}}}{\partial w_{pq}},\;p,q\in \left\{E,I\right\}$$

In the process of adaptation of biophysical parameters, the four coupling parameters of the WC module are iteratively optimized under the guidance of the physical residuals, so that the learned evolution of E/I states becomes more consistent with the imposed dynamic constraints of the WC. With iterative training, the learned coupling parameters exhibit systematic trends that are consistent with known neurophysiological patterns related to E/I. Specifically, the observed changes include increased excitatory self-activation ($W_{EE}$), weakened inhibitory regulation of excitation ($W_{EI}$), enhanced excitation-driven inhibition ($W_{IE}$), and reduced inhibitory self-inhibition ($W_{II}$). These directional changes are consistent with excitatory enhancement and inhibitory imbalance, which are frequently reported during transitions between epileptic states.

## 3. E/I-DynPolyNet-Based Method for EEG Epilepsy Diagnosis

### 3.1. Overall Framework

**Figure 3:**
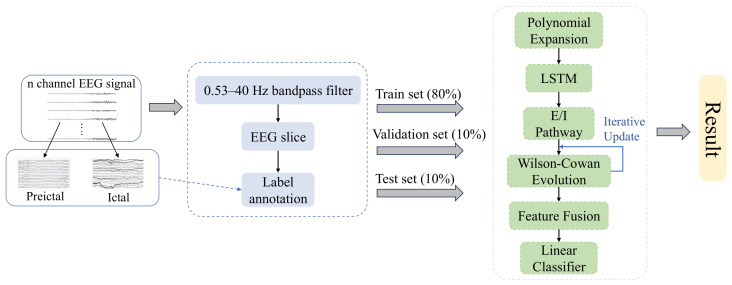
Workflow of epilepsy diagnosis based on E/I-DynPolyNet.

Under the introduced E/I-DynPolyNet model, we present an epilepsy seizure detection methodology utilizing multi-channel EEG signals, as illustrated in the workflow diagram shown in Figure 3. The diagnostic procedure comprises three principal stages: (1) data acquisition and preprocessing, (2) feature extraction, and (3) seizure state classification based on the E/I-DynPolyNet model.

Before temporal encoding, the lightweight polynomial expansion is applied to the time-series EEG amplitudes. By appending element-wise quadratic terms to the temporal input sequence, the model avoids constructing dense pairwise cross-channel interactions, thereby reducing the risk of misinterpreting isolated high-amplitude fluctuations as physiologically meaningful nonlinear patterns. Initial data extraction from multi-channel EEG recordings of epilepsy patients consists of two steps: preprocessing and labeling. During the preprocessing step, pre-ictal and ictal onset labels are recorded as raw input for the model. Bandpass filtering (0.5–40 Hz) is applied to remove physiological artifacts and high-frequency noise while preserving the spectral components that are most useful for epilepsy activity. Then, EEG slices are created and labeled by seizure state.

In order to classify seizure state, the fused feature representation is mapped to decision space by a dual-layer MLP classifier, followed by softmax normalization to obtain probabilistic category prediction. The E/I-DynPolyNet model is trained end-to-end using AdamW, a decoupled weight decay variant of Adaptive Moment Estimation (Adam). Unlike the traditional Adam optimizer, AdamW provides more stable convergence [26], particularly when handling dynamic residual terms with multi-scale gradients, which is important for maintaining the stability of the WC parameters during long-term temporal encoding. This is coupled with a cosine annealing scheduler with warmup, which can balance multi-scale gradient magnitudes in neurodynamic modeling tasks [27]. The optimization objective employs a composite loss function, integrating cross-entropy loss from classification supervision with a physical information regularization term. The training and testing phases are executed through the utilization of 10-fold cross-validation. Upon completion, the E/I-DynPolyNet model provides a quantitative assessment through the excitation/inhibition ratio metric, alongside binary classification results.

### 3.2. Data Acquisition and Preprocessing

Raw continuous EEG signals are segmented into fixed-length windows:

(38) $$X_{\mathrm{raw}}\in R^{N_{\mathrm{win}}\times T\times C},$$

where $N_{\mathrm{win}}$ denotes the number of extracted EEG windows, *T* is the temporal length of each window, and *C* represents the number of EEG channels. In this study, each EEG window contains T=512 time points. Each window is assigned a binary label according to the seizure annotation, where the pre-ictal state is labeled as 0 and the ictal state is labeled as 1.

A data cleaning phase is implemented to enhance data quality. First, any samples that contain empty values are replaced with zeros to ensure numerical stability. Subsequently, an Isolation Forest algorithm is employed with a contamination factor of 0.01 to automatically detect and remove anomalous windows or artifacts that deviate significantly from the typical neural oscillatory patterns. For each cross-validation fold, the Isolation Forest and normalization statistics are fitted only on the training subset and then applied to the validation and test subsets to avoid information leakage.

To preserve subject-specific patterns, windows are organized at the session level. For session *s* containing $n_{s}$ windows, when $n_{s}$ is odd, the middle transition trial at index $m=\left\lfloor n_{s}/2\right\rfloor$ is removed. This ensures balanced class distribution: $n_{\mathrm{preictal}}=n_{\mathrm{ictal}}=n_{s}^{\mathrm{final}}/2$.

Then, channel-wise z-score normalization is applied as follows:

(39) $$x_{it}=\frac{x_{it}-\mu_{i}}{\sigma_{i}},$$

where $\mu_{i}$ and $\sigma_{i}$ are the mean and standard deviation of the channel *i* computed across all training samples, respectively.

### 3.3. Epilepsy Diagnosis Framework

After data preprocessing and normalization, the preprocessed EEG sequences are fed into the E/I-DynPolyNet model for epilepsy state classification. The model is configured with a polynomial expansion order of 2 and an initial learning rate of 0.0004, and all weight matrices ($W_{E},W_{I1},W_{I2}$) are initialized via Xavier uniform initialization. The WC coupling coefficients ($W_{EE}$, $W_{EI}$, $W_{IE}$, $W_{II}$) are initialized under biologically motivated constraints. Following the WC population model, these coefficients are treated as non-negative effective interaction strengths between excitatory and inhibitory neural populations [23]. Accordingly, their unconstrained raw variables are initialized near zero and mapped to positive values using the Softplus transformation:

(40) $$W_{pq}=\mathrm{Softplus}\left(W_{pq}^{raw}\right),\;p,q\in \left\{E,I\right\}.$$

Inhibitory effects are represented by the subtractive structure of the WC equations, rather than by assigning negative values to inhibitory coupling coefficients. The excitatory and inhibitory time constants are also constrained to positive ranges. These settings provide biologically interpretable initial constraints and stable starting points, while all WC-related coefficients remain trainable during optimization.

The E/I imbalance is a dynamic neurophysiological phenomenon, and its characteristics are manifested in the temporal evolution of EEG signals. To capture the dynamically evolving pattern of E/I imbalance, E/I-DynPolyNet adopts a causal temporal encoder to extract temporal features and model the dynamic process of E/I imbalance. In preliminary experiments, we attempted to replace the causal temporal encoder with a self-attention-based temporal encoder. However, the results showed that this replacement led to a substantial increase in the physical residuals. This may be because unrestricted self-attention allows information from future time steps to affect the representation of the current time step, which is not fully consistent with the causal evolution assumed in E/I dynamics. Therefore, LSTM was adopted as the main causal temporal encoder. Attention was used only in the later hybrid temporal pooling stage to aggregate the encoded sequence together with mean, maximum, and last-state features.

The E/I pathway mechanism, which supports the E/I-DynPolyNet model, mimics excitatory and inhibitory neuron groups in cortical microcircuits. By doing so, it provides an interpretable way to model the E/I imbalance associated with epilepsy. The module processes temporal-domain EEG features through the implementation of dedicated nonlinear activation functions and weight-constrained independent pathways. This facilitates the capture of the asymmetric dynamics between excitation and inhibition, thereby enabling interpretable modeling of epileptic E/I imbalance.

**Figure 4:**
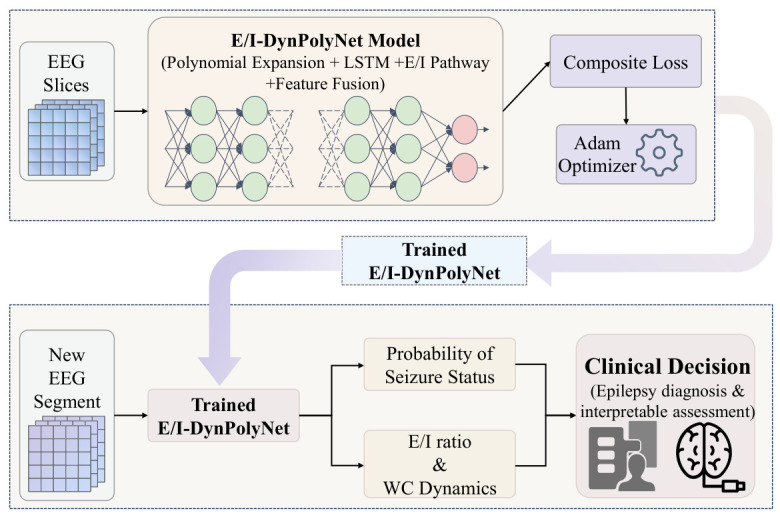
Detailed training pipeline of E/I-DynPolyNet for EEG-based seizure diagnosis.

The process of diagnosing epilepsy using E/I-DynPolyNet is shown in Figure 4. During training, the AdamW optimizer updates model parameters using Adaptive Moment Estimation, and the learning rate is managed by a cosine annealing scheduler with a 6-epoch warmup phase, with the base learning rate initially set to 0.0004 for the neural backbone and 0.00002 for the biophysical parameters to ensure stable convergence. Gradient clipping constrains the L2-norm to 1.0 to prevent instability during the WC evolution. The composite loss function L_total_ combines cross-entropy loss, physics residual loss ($\lambda_{\mathrm{phy}}=0.02$), E/I balance loss ($\lambda_{\mathrm{bal}}=0.005$), and L2 regularization ($\lambda_{\mathrm{reg}}=0.0001$). Training proceeds with a batch size of 64, terminating after 100 epochs or when the validation accuracy fails to improve for 35 consecutive epochs.

Upon completion of training, the model is evaluated in a 10-fold stratified setting, where each fold tests on held-out samples. Test EEG signals undergo the same preprocessing pipeline and are fed into the trained model to produce class probabilities via a softmax layer. Performance is reported using accuracy, precision, recall, F1-score, physics residual, and E/I ratio.

## 4. Experimental Results

### 4.1. Experimental Datasets and Environment

This study employs two benchmark EEG databases to evaluate the proposed method: the publicly available CHB-MIT scalp EEG database, and the single-channel EEG dataset released by the University of Bonn [28].

The CHB-MIT database contains multi-session scalp EEG recordings from pediatric subjects diagnosed with epilepsy. All signals are resampled to 256 Hz to ensure comparability across sessions. For analysis, we adopted a fixed 16-channel bipolar montage, defined as FP1-F7, F7-T7, T7-P7, P7-O1, FP1-F3, F3-C3, C3-P3, P3-O1, FP2-F4, F4-C4, C4-P4, P4-O2, F8-T8, Fz-Cz, Cz-Pz, and FP2-F8. The continuous EEG recordings were segmented into 2 s windows with a 1 s step size, corresponding to 512 time points per window and a 50% overlap between adjacent windows. Each window was assigned a binary label according to the seizure annotation. The resulting data instances are represented as [N,T,C], where *N* is the number of windows, *T* denotes the time point, and *C* represents the channel.

To verify the model’s versatility, we incorporated the Bonn dataset, which consists of single-channel EEG segments. The Bonn data used in this study are provided in a feature-based form, where each EEG segment is represented by 24 extracted descriptors. These descriptors summarize segment-level EEG characteristics rather than raw temporal samples or spatial EEG channels.

Following the unified tensor convention [N,T,C], the Bonn samples were organized as [N,1,24], where N=200 denotes the number of EEG segments, T=1 denotes one feature vector for each segment, and C=24 denotes the extracted feature descriptors. After the anomaly detection step, 198 samples were retained for model evaluation, as summarized in Table 1. This format was used only to maintain compatibility with the model input interface. The 24 features were not treated as a temporal sequence; instead, they were encoded as a compact segment-level representation before being projected into latent E/I states. Therefore, for the Bonn dataset, the WC module constrains the learned latent E/I representation rather than directly modeling the raw temporal evolution of EEG signals.

To ensure the numerical stability of the physics-informed constraints, we implemented an unsupervised anomaly detection step using the Isolation Forest algorithm. A contamination rate of ν=0.01 was set to identify and remove the top 1% of samples exhibiting statistical anomalies or extreme physiological artifacts, significantly reducing gradient instability during the solution of differential equations. This procedure was conducted within each cross-validation fold: the Isolation Forest model was fitted only on the training subset and then applied to the corresponding validation and test subsets without refitting.

For CHB-MIT, subject-level grouped 10-fold cross-validation was adopted, and all windows from the same subject were assigned exclusively to one fold. This setting prevented both subject-level leakage and the splitting of adjacent or overlapping windows from the same recording across training and test subsets. Within each fold, 10% of the training samples were used for validation, and Z-score normalization was fitted only on the training subset before being applied to the validation and test subsets. Table 1 summarizes the class distribution after data segmentation.

All experiments were implemented in PyTorch v2.0.1 on an NVIDIA GeForce RTX 4090 GPU (NVIDIA Corporation, Santa Clara, CA, USA) with automatic mixed precision. The model used a two-layer LSTM encoder with a hidden size of 160, an 80-dimensional E/I latent representation, eight WC evolution steps, and four-head temporal attention pooling. The conditional WC generator used compact hidden layers of 64/32 for the coupling branch and 16 for the time-constant branch, followed by a 128-dimensional gated fusion module and a classifier with a 64-dimensional hidden layer. Layer normalization was adopted instead of batch normalization, with dropout rates of 0.15, 0.25, 0.35, and 0.20 in the E/I pathways, E/I post-processing layers, fusion module, and classifier, respectively. AdamW was configured with $\beta_{1}=0.9$, $\beta_{2}=0.999$, $\epsilon =10^{-8}$, a backbone learning rate of $4\times 10^{-4}$, a WC-parameter learning rate of $2\times 10^{-5}$, and a weight decay of $2\times 10^{-4}$. The computational profile of the model is summarized in Table 2. During test-time dynamical alignment, predictions were averaged over WC evolution steps of 3, 5, and 8.

The computational scalability of E/I-DynPolyNet is mainly determined by the sequence length *T*, the LSTM hidden dimension *H*, the E/I latent dimension *D*, and the number of WC evolution steps *K*. The lightweight polynomial expansion only doubles the input channel dimension from *C* to 2C, avoiding the quadratic growth caused by full pairwise polynomial expansion. The LSTM encoder scales approximately linearly with the sequence length, while the WC dynamic module introduces an additional linear factor with respect to *K*. Therefore, increasing the EEG window length or the number of WC steps increases the computational cost in an approximately linear manner. In contrast, the temporal attention pooling module uses a learnable query for sequence aggregation rather than full pairwise self-attention, which limits the additional cost of temporal aggregation.

### 4.2. Parameter Sensitivity and Stability Analysis

**Figure 5:**
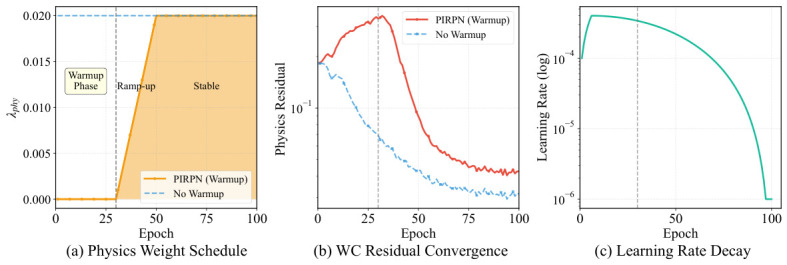
Convergence dynamics and optimization stability analysis of E/I-DynPolyNet.

To balance data-driven feature representation with biophysical law constraints, we implemented a three-stage weight scheduling strategy. As illustrated in Figure 5a, $\lambda_{\mathrm{phy}}$ was maintained at 0 to prioritize spatiotemporal feature extraction during the warmup phase. This is followed by a ramp-up phase, where $\lambda_{\mathrm{phy}}$ linearly increases to 0.02, before finally reaching a stable phase to establish equilibrium between classification accuracy and biological interpretability. The comparative analysis in Figure 5b illustrates that the baseline without warmup suffers from local optima and conflicting constraints, while warmup mode demonstrates a stable exponential decay in WC residuals, eventually converging below $10^{-1}$. As shown in Figure 5c, the learning rate peaks at $4\times 10^{-4}$ during the feature exploration stage and smoothly decays to $10^{-6}$ during the stable phase. This reveals that, without disrupting the established E/I balance, E/I-DynPolyNet achieves a refinement of seizure classification boundaries.

### 4.3. E/I Imbalance Visualization

**Figure 6:**
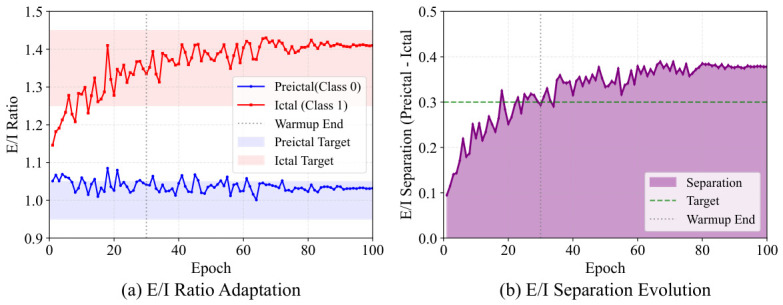
Evolution of E/I ratio and E/I separation during training.

On the validation set, the E/I-DynPolyNet model demonstrates well-behaved convergence of the E/I ratio along with physiologically motivated latent dynamics. As shown in Figure 6a, the overall E/I ratio starts at approximately 1.15 for the ictal state and 1.05 for the pre-ictal state. After minor fluctuations during the early phase of training, the ratios stabilize after the warmup period, eventually converging toward the target ranges. This stabilization occurs in parallel with the convergence of classification accuracy, suggesting that the E/I-related representation learned by E/I-DynPolyNet forms a stable latent pattern rather than being solely driven by random fluctuations.

The pre-ictal and ictal states exhibit a persistent and clearly separable pattern along the E/I axis, consistent with commonly reported excitation-dominant patterns during seizures. As illustrated in Figure 6a, the pre-ictal trajectory remains relatively stable throughout training, with E/I ratios ranging from 1.05 to 1.03. In contrast, the ictal trajectory consistently exceeds the pre-ictal levels, with values spanning 1.15 to 1.43 and converging toward approximately 1.41 in the later phase.

Further analysis of the E/I separation evolution in Figure 6b demonstrates that the distance between the two classes grows steadily from an initial 0.1 to nearly 0.4 by the end of training. This separation quickly surpasses the target threshold of 0.3 shortly after the warmup phase.

For each fold, the E/I difference was calculated as the mean E/I ratio of ictal samples minus that of pre-ictal samples, and the resulting fold-wise differences were tested against zero using a one-sample *t*-test. As summarized in Table 3, the E/I difference was 0.3420±0.0101, with a 95% confidence interval of [0.3348, 0.3492]. The interval was entirely above zero, and the difference was statistically significant. Cohen’s $d_{z}$ was 33.86, indicating that the E/I difference was highly consistent relative to its cross-fold variability. These results suggest that the learned E/I-related latent representation provides a stable separation between pre-ictal and ictal states. However, this E/I ratio should be interpreted as a model-derived latent indicator rather than a direct measurement of biological excitatory or inhibitory neuronal activity.

**Figure 7:**
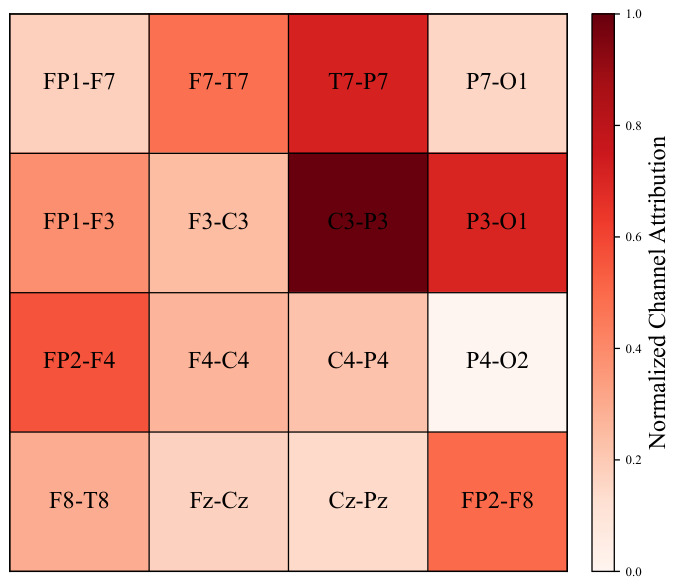
Seizure-onset E/I attribution across channels.

To further examine channel-wise interpretability, Figure 7 visualizes the seizure-onset minus pre-ictal attribution shift of the learned E/I representation across the 16 EEG channels, showing that the attribution shift is not uniformly distributed across channels. Relatively stronger responses are observed in C3-P3, T7-P7, and P3-O1, suggesting channel-dependent heterogeneity in the learned E/I-related representation during the seizure-onset stage. The attribution values were min–max normalized for visualization. This channel-level pattern reflects model-derived attribution to the learned E/I representation, rather than clinical seizure-focus localization.

### 4.4. Classification Results

The E/I-DynPolyNet model was evaluated on the CHB-MIT and Bonn datasets using 10-fold cross-validation. The model was trained using the AdamW optimizer with EEG segments or feature-based EEG representations as inputs. A residual physical constraint term was applied throughout the training to encourage consistency with the imposed WC-based latent dynamics.

Table 4 reports the overall performance, and the E/I difference is included as an additional quantitative index of E/I imbalance in the learned representations. On CHB-MIT, E/I-DynPolyNet achieves an accuracy and an F1-score of 95.81%±0.8%, with an E/I difference of 0.34. The results demonstrate effective seizure state classification and more pronounced E/I-related differences that are consistent with physiologically motivated E/I patterns, supporting the model’s interpretability. In contrast, the Bonn dataset shows a more minor E/I difference of 0.14, while E/I-DynPolyNet still attains a high accuracy of 98.5%. The smaller E/I difference on Bonn may be related to its single-channel setting. Unlike CHB-MIT, which provides multi-channel spatial information for identifying localized E/I imbalance and inter-channel seizure propagation, Bonn only reflects a one-dimensional temporal projection of EEG activity. As a result, the WC constraint on Bonn may primarily serve as a temporal regularizer, whereas on CHB-MIT it can more effectively capture physiologically meaningful E/I coupling patterns.

**Figure 8:**
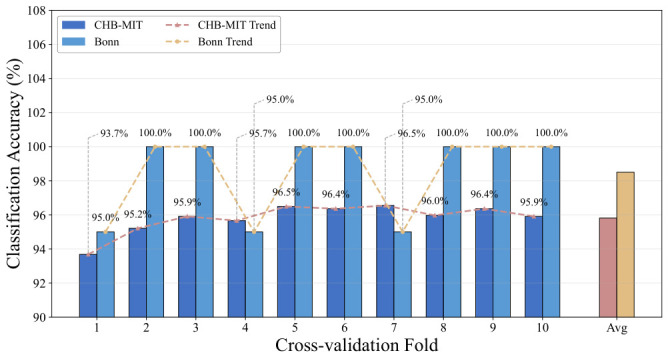
Classification accuracy on the CHB-MIT and Bonn datasets.

Figure 8 reports the 10-fold test accuracy of E/I-DynPolyNet on the CHB-MIT and Bonn datasets. On CHB-MIT, the accuracy ranges from 93.7% to 96.5%, with a standard deviation of 0.8%. On Bonn, the accuracies range from 95.0% to 100%. More importantly, the accuracies show limited variation across folds on both datasets, as well as no pronounced degradation in any individual fold, suggesting stable performance across different splits. This stability is consistent with the model design. The causal temporal encoder captures temporal dependencies, while the E/I separation decomposes the temporal features into complementary E/I pathways, providing a physiologically meaningful representation for seizure state discrimination. In addition, the WC dynamics serves as a physics-based regularizer, constraining the learned E/I evolution to follow the WC dynamical system. Together, these components promote more consistent solutions across sessions and recording conditions, resulting in stable accuracy. The averaged results on the right further confirm that E/I-DynPolyNet achieves competitive and consistent performance on both benchmarks.

**Figure 9:**
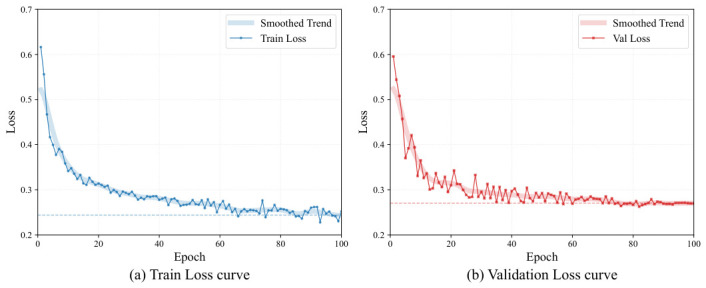
Training and validation loss in CHB-MIT dataset.

Figure 9 shows the training and validation loss curves of E/I-DynPolyNet on the CHB-MIT dataset. The training loss decreases rapidly in the early epochs, dropping from around 0.60 to below 0.3 within approximately 30 epochs, and then continues to decline smoothly to about 0.23–0.25. The validation loss exhibits a similar downward trend, falling from 0.6 to around 0.3 in the first 30 epochs and then remaining stable, with only slight fluctuations across folds. The validation loss tracks the training loss closely, reflecting stable generalization performance and reduced overfitting. We attribute this behavior to the physics-informed constraint induced by the WC dynamics, which introduces a structured inductive bias and regularizes the solution space. In addition, the multi-level dropout strategy and the proposed E/I balance loss further mitigate overfitting, resulting in consistent validation performance in the late training stage and supporting the effectiveness of E/I-DynPolyNet for seizure detection.

## 5. Performance Comparison and Experimental Evaluation

### 5.1. Ablation Experiments

To quantify the contribution of each component to overall performance, we performed a series of ablation studies, and the results are summarized in Table 5. The full E/I-DynPolyNet model yielded the best mean accuracy of 95.81%±0.8%, validating the effectiveness of the integrated design. When the polynomial expansion was removed, only marginal performance degradation was observed, suggesting that these two components primarily contribute to the representational capacity of the features and the stability of the model. Subsequently, when the E/I separation was eliminated, the accuracy drastically dropped to 92.54%. This suggests that the module is not merely an architectural component but actually operates on the temporal features extracted by the causal temporal encoder, decomposing them into two complementary pathways to model the key mechanism of E/I imbalance. Without this module, the model fails to capture this physiological signature, resulting in a significant loss of interpretability. The absence of the causal temporal encoder caused a substantial decline to 91.33%, confirming that temporal dependency modeling is indispensable for capturing the dynamic evolution of E/I imbalance. Overall, the Taylor expansion increases nonlinear expressiveness, the causal temporal encoder captures long-range temporal dependencies, E/I separation obtains physiologically meaningful dual-pathway representations, and the WC dynamics restrict their interaction and evolution. This combination of data-driven learning and physics-based constraint not only restores the accuracy but, with the learned representations consistent with neural dynamics, achieves a balance between performance and interpretability.

### 5.2. Comparative Experiments

To systematically assess the performance of the proposed E/I-DynPolyNet, we conducted a comprehensive comparison against traditional machine learning, state-of-the-art (SOTA) deep learning, and recent physics-informed frameworks. The benchmark methods included the following: traditional machine learning: Random Forest; graph neural networks and spatiotemporal graph models: Graph Dynamic Network (GDN) and Graph Temporal Attention (GTA); representative SOTA deep learning methods: CNN-LSTM [29] and Dynamic Temporal-Spatial Graph Attention Network (DTS-GAN) [30]; recent physics-informed and neurodynamical frameworks: HP-GNN, which incorporates Kuramoto oscillator dynamics within a hypergraph neural network, and a Neural ODE-enhanced framework [31] that models EEG signals as continuous-time dynamical systems; and the proposed method: E/I-DynPolyNet.

It should be noted that, due to variations in experimental protocols across the literature, HP-GNN results are reported as the mean SD over 4-fold cross-validation, while Neural ODE-enhanced results represent an average performance across multiple datasets, including CHB-MIT. In contrast, all traditional and deep learning baselines, as well as our proposed E/I-DynPolyNet, were evaluated using 10-fold stratified cross-validation to ensure a consistent and rigorous comparison.

Table 6 provides a summary of the quantitative results for all evaluated methods on the test set. The experimental results show that E/I-DynPolyNet achieves competitive predictive performance while offering neurophysiological interpretability. Compared to traditional machine learning and earlier graph-based networks (GDN and GTA), E/I-DynPolyNet demonstrates a significant improvement in accuracy and sensitivity, suggesting that incorporating neurobiological constraints into model design provides a superior inductive bias for EEG anomaly detection.

Among physics-informed frameworks, E/I-DynPolyNet shows favorable mechanistic consistency. While Neural ODE frameworks achieve high reported accuracy by modeling EEG as continuous-time dynamical systems, they typically rely on flexible black-box parameterizations and do not explicitly impose neurophysiological E/I constraints. In contrast, E/I-DynPolyNet embeds the Wilson–Cowan equations into the learning framework, allowing hidden states to be associated with quantifiable E/I population interactions. Although the reported HP-GNN- and Neural ODE-enhanced results are not strictly comparable due to differences in evaluation protocols, they provide useful references for positioning E/I-DynPolyNet among recent neurodynamical and physics-informed EEG learning methods. Overall, the proposed model achieves competitive classification performance while retaining an explicit E/I-based interpretability mechanism. To further evaluate the statistical reliability of the improvement over the strongest comparable baseline, we conducted paired statistical testing between E/I-DynPolyNet and CNN-LSTM using the 10-fold cross-validation results. E/I-DynPolyNet achieved a mean accuracy improvement of 1.43 percentage points over CNN-LSTM. The paired *t*-test indicated a significant difference, with t(9)=5.29 and $p=5.0\times 10^{-4}$. The Wilcoxon signed-rank test also confirmed this improvement, with W=1.0 and $p=3.91\times 10^{-3}$. The corresponding paired effect size was large, with Cohen’s $d_{z}=1.67$, suggesting that the performance gain is statistically significant and practically meaningful.

**Figure 10:**
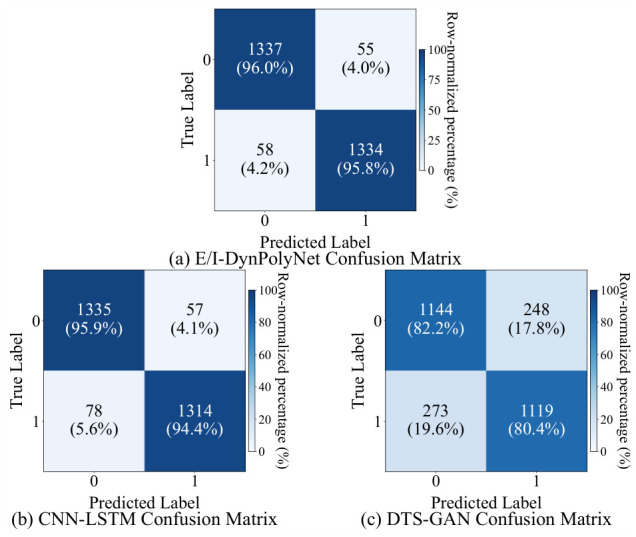
Confusion matrices for three models.

To further illustrate the classification behavior of E/I-DynPolyNet, Figure 10 presents the confusion matrices of CNN-LSTM and DTS-GAN alongside the proposed model. The percentages in each cell are normalized within the corresponding true class, while the raw sample counts are also retained for reference. CNN-LSTM misclassifies 78 epileptic samples as normal, whereas E/I-DynPolyNet reduces this number to 58. The proposed model also maintains slightly fewer false alarms for normal samples, with 55 normal samples misclassified as epileptic, compared with 57 for CNN-LSTM. Compared with DTS-GAN, E/I-DynPolyNet shows a more evident reduction in misclassifications for both categories. These results suggest that the proposed model achieves more balanced discrimination between the two categories, particularly in reducing missed epileptic detections.

## 6. Conclusions

We propose the E/I-DynPolyNet model for identifying epileptic states through EEG signals based on biophysical dynamics principles. By integrating polynomial expansions with dual E/I pathways, the network introduces a nonlinear yet interpretable transformation that aligns feature evolution with the equilibrium behavior of neural population dynamics. The residual polynomial mapping and the time-domain gating module derived from WC dynamics adaptively regulate temporal information flow, mitigating vanishing gradients and encouraging causal consistency implied by the underlying dynamics. Standard residual connections further facilitate stable feature propagation, improving optimization robustness and enhancing representational capacity. Furthermore, adaptive learning-rate scheduling and gradient normalization accelerate convergence while balancing fast excitatory and slower inhibitory responses during training. While higher-order polynomial terms can enrich local feature interactions, their marginal gains are smaller than those of the temporal and biophysical components, and they introduce additional computational cost in high-dimensional settings. Future research will explore lightweight polynomial formulations and efficient regularization strategies to enhance scalability further.

## Figures and Tables

**Table 1:** Distribution of epilepsy labels and data segmentation.

Class	Dataset Size		Train/Validation Ratio
	CHB-MIT Dataset	Bonn Dataset	
Pre-ictal (0)	7907 (50.5%)	100 (50.5%)	Train 90%/Validation 10%
Ictal (1)	7748 (49.5%)	98 (49.5%)	

**Table 2:** Computational profile of E/I-DynPolyNet on CHB-MIT.

Item	Value
Random seed	3407
Input window size	512×16
Batch size	64
Trainable parameters	2.60 M
FLOPs per forward pass	0.86 GFLOPs
FLOPs with TTA	2.28 GFLOPs
Inference time	0.4 ms/window

**Table 3:** Statistical analysis of E/I ratio difference on the CHB-MIT dataset.

E/I Diff.	95% CI	*t*-Value	*p*-Value	Effect Size
0.3420 ± 0.0101	[0.3348, 0.3492]	107.1	*p* < 1 × 10^−5^	$d_{z}=33.86$

**Table 4:** Overall performance on CHB-MIT and Bonn datasets.

Dataset	Acc. (%)	F1 (%)	E/I Diff.
CHB-MIT	95.81±0.8	95.81±0.8	0.34±0.1
Bonn	98.5±2.4	98.5±2.4	0.14±0.16

**Table 5:** Ablation results of E/I-DynPolyNet in the CHB-MIT dataset.

Model	Accuracy	Std Dev
Full Model	95.81%	±0.8%
w/o Poly.	93.68%	±1.2%
w/o Encoder	91.33%	±1.6%
w/o E/I	92.54%	±0.6%

**Table 6:** Performance comparison for EEG seizure detection on CHB-MIT dataset.

Method	Category	Accuracy	Sensitivity	Specificity
Traditional/Deep Learning Baselines				
Random Forest	Traditional ML	73.98%	72.11%	75.84%
GDN	Graph Neural	71.58%	68.92%	74.25%
GTA	Graph Neural	78.56%	76.26%	80.86%
CNN-LSTM [29]	Hybrid	94.38%	94.38%	95.89%
DTS–GAN [30]	Graph + LSTM	80.39%	80.39%	82.19%
Physics-Informed/Neurodynamical				
HP-GNN *	Phys.-Inf. GNN	84.7%	89.3%	80.1%
Neural ODE-Enhanced [31] *	NODE-based Dynamics	98.2%	97.8%	98.3%
E/I-DynPolyNet (Ours)	Physics-Informed (E/I ratio quantifiable)	95.81%	95.81%	96.08%

Note: * Results are taken from the original reports under different evaluation protocols: HP-GNN used 4-fold cross-validation, while the Neural ODE-enhanced framework reported averaged results across multiple datasets. Thus, they are provided only as contextual references.

## Data Availability

The original CHB-MIT scalp EEG dataset and Bonn EEG dataset are publicly available from their respective data repositories. The processed data and experimental records used in this study will be made available by the corresponding author upon reasonable request. The source code, model implementation, preprocessing scripts, and training configuration are available from the corresponding author upon reasonable request.
